# Modeling of ACE2 and antibodies bound to SARS-CoV-2 provides insights into infectivity and immune evasion

**DOI:** 10.1172/jci.insight.168296

**Published:** 2023-07-10

**Authors:** Joseph H. Lubin, Christopher Markosian, D. Balamurugan, Minh T. Ma, Chih-Hsiung Chen, Dongfang Liu, Renata Pasqualini, Wadih Arap, Stephen K. Burley, Sagar D. Khare

**Affiliations:** 1 Department of Chemistry and Chemical Biology, Rutgers, The State University of New Jersey, Piscataway, New Jersey, USA.; 2 Rutgers Cancer Institute of New Jersey, Newark, New Jersey, USA.; 3 Division of Cancer Biology, Department of Radiation Oncology, Rutgers New Jersey Medical School, Newark, New Jersey, USA.; 4 Office of Advanced Research Computing, Rutgers, The State University of New Jersey, Piscataway, New Jersey, USA.; 5 Department of Radiology,; 6 Department of Pathology, Immunology, and Laboratory Medicine,; 7 Center for Immunity and Inflammation, and; 8 Division of Hematology/Oncology, Department of Medicine, Rutgers New Jersey Medical School, Newark, New Jersey, USA.; 9 RCSB Protein Data Bank, Rutgers, The State University of New Jersey, Piscataway, New Jersey, USA.; 10 RCSB Protein Data Bank, San Diego Supercomputer Center, UCSD, La Jolla, California, USA.; 11 Rutgers Cancer Institute of New Jersey, New Brunswick, New Jersey, USA.; 12 Institute for Quantitative Biomedicine, Rutgers, The State University of New Jersey, Piscataway, New Jersey, USA.

**Keywords:** COVID-19, Bioinformatics, Immunoglobulins, Structural biology

## Abstract

Given the COVID-19 pandemic, there is interest in understanding ligand-receptor features and targeted antibody-binding attributes against emerging SARS-CoV-2 variants. Here, we developed a large-scale structure-based pipeline for analysis of protein-protein interactions regulating SARS-CoV-2 immune evasion. First, we generated computed structural models of the Spike protein of 3 SARS-CoV-2 variants (B.1.1.529, BA.2.12.1, and BA.5) bound either to a native receptor (ACE2) or to a large panel of targeted ligands (*n* = 282), which included neutralizing or therapeutic monoclonal antibodies. Moreover, by using the Barnes classification, we noted an overall loss of interfacial interactions (with gain of new interactions in certain cases) at the receptor-binding domain (RBD) mediated by substituted residues for neutralizing complexes in classes 1 and 2, whereas less destabilization was observed for classes 3 and 4. Finally, an experimental validation of predicted weakened therapeutic antibody binding was performed in a cell-based assay. Compared with the original Omicron variant (B.1.1.529), derivative variants featured progressive destabilization of antibody-RBD interfaces mediated by a larger set of substituted residues, thereby providing a molecular basis for immune evasion. This approach and findings provide a framework for rapidly and efficiently generating structural models for SARS-CoV-2 variants bound to ligands of mechanistic and therapeutic value.

## Graphical abstract

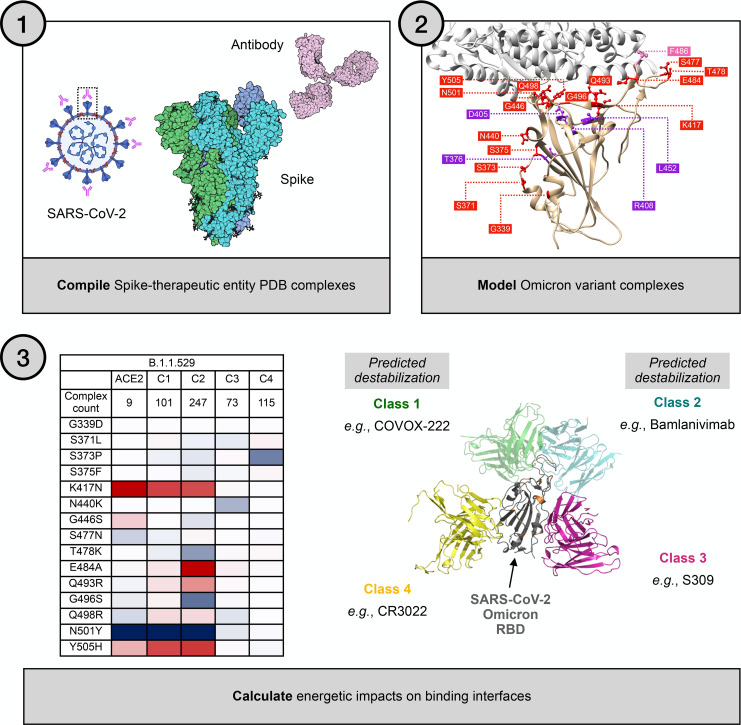

## Introduction

**Figure 1:**
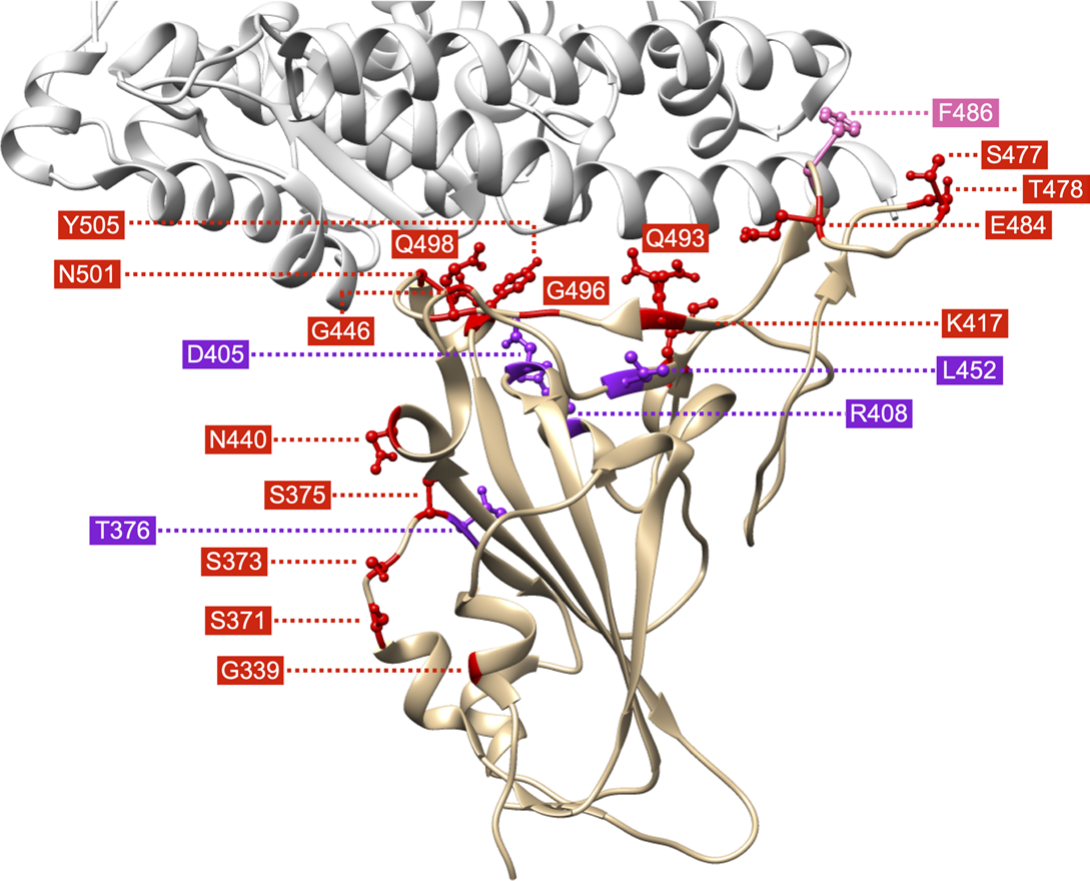
Overview of residues of Spike of SARS-CoV-2 that are substituted in the RBD (tan) of the original Omicron VOC (B.1.1.529) and subvariants BA.2.12.1 and BA.5 (see Table 1). Residue sites are colored according to mutation appearance by subvariant (red for B.1.1.529, purple for BA.2.12.1, and pink for BA.5). RBD contains the RBM, which interacts with ACE2 (light gray) (PDB ID: 6M0J).

The Omicron variant of SARS-CoV-2, first sequenced in the Republic of South Africa as B.1.1.529, was deemed a variant of concern (VOC) by the World Health Organization (WHO) and its subvariants are currently spreading among humans worldwide (1). During the initial discovery of this variant, relatively little was known about its transmissibility, potential for immune evasion, and virulence (2, 3). However, a distinguishing feature of this VOC and its subvariants is the large number of amino acid residue changes detected in the Spike protein (hereafter referred to as Spike) versus all previously characterized coronaviral strains. Many of these residue substitutions map to the receptor-binding domain (RBD) (Figure 1 and Table 1), which is consistent with their dampening or evasion of individual immune responses generated from vaccination and/or previous infection. Immune evasion may have serious consequences, potentially leading to increased incidence of (re)infection and/or further enhancing viral fitness during evolution in the event of uncontrolled global spread. Findings reveal substantial (but not complete) immune evasion of the Omicron VOC and its subvariants to an even higher degree (4–7). A second concern has been that biological therapeutics, such as monoclonal antibody combinations or nanobodies, developed against previously characterized SARS-CoV-2 variants may no longer be effective in neutralizing the Omicron VOC and its subvariants, ultimately leading to increased morbidity and mortality. Advanced knowledge of the immune evasion properties of an emerging variant would help in developing novel pharmaceutical and nonpharmaceutical interventions against the spread of SARS-CoV-2.

At the core of concerns regarding immune evasion is molecular recognition in 3 dimensions (3D) between the Spike and ligands, encompassing at least one established native cellular receptor, angiotensin-converting enzyme 2 (ACE2), neutralizing antibodies of natural origin, engineered therapeutic antibodies, and other binding proteins. Several fundamental questions must be addressed. First, is binding of these agents to Spike of the Omicron VOC or its subvariants substantially altered versus previous variants of SARS-CoV-2 (e.g., the Delta VOC or the original wild-type, Wuhan-Hu-1, strain)? Second, do the numerous residue changes within the Spike substantially alter its shape (3D structure)? If so, do these changes lead to a meaningful remodeling of interfaces formed with various binding proteins? Finally, are these remodeled interfaces likely to weaken recognition of Spike by some or all of the neutralizing ligands and do these molecular changes correlate with neutralization data? The ability to answer these questions before a given variant sweeps the human population would likely have profound impact in biology, medicine, and public health policy in the setting of an ongoing global airborne pandemic.

**Figure 2:**
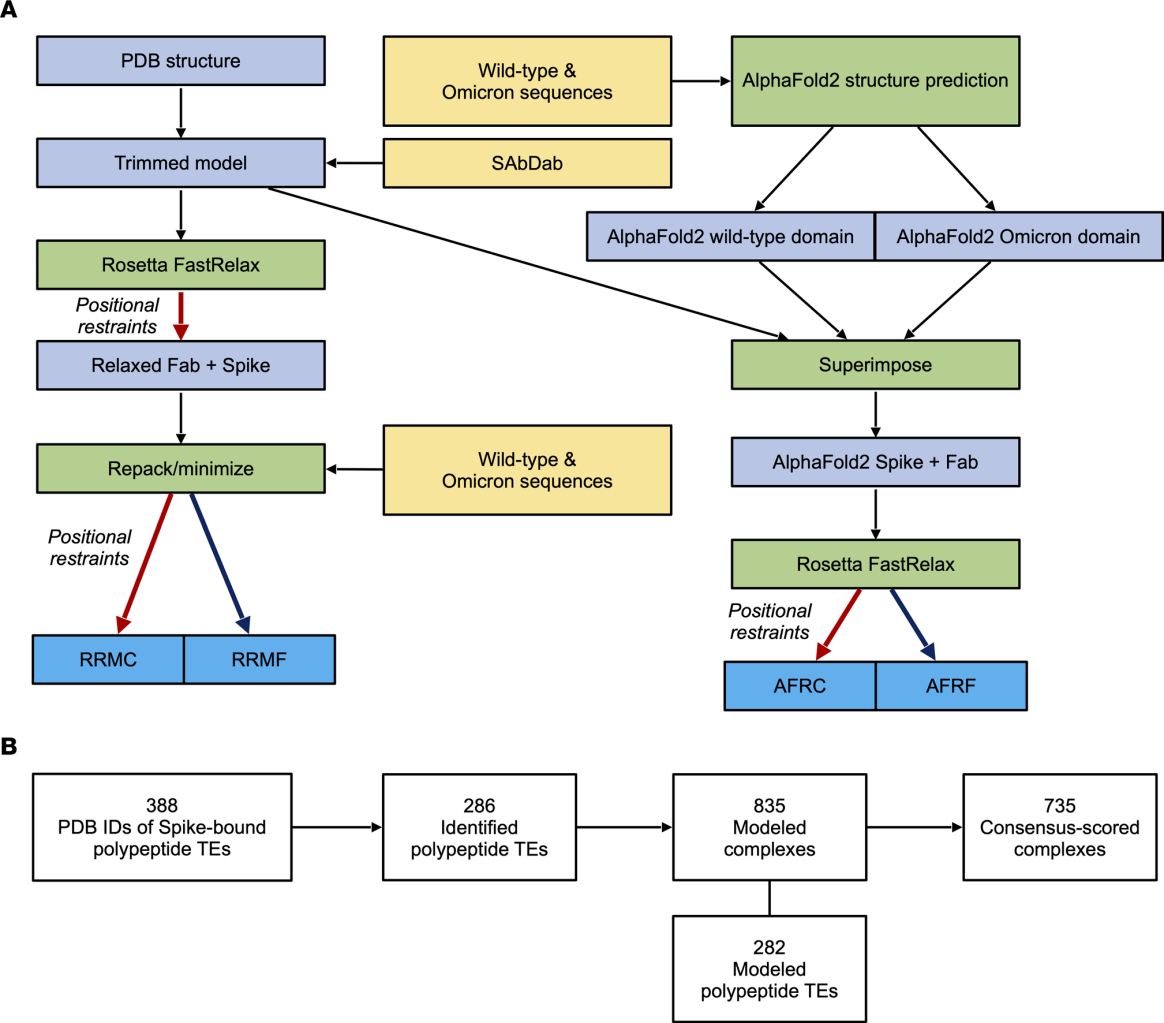
Methodology utilized to evaluate the effects of Omicron variant mutations on polypeptide binding to Spike. (**A**) Workflow to generate predicted structures of Spike of B.1.1.529, BA.2.12.1, and BA.5 in complex with various polypeptide TEs, including antibodies, based on experimentally solved structures. (**B**) Overall breakdown of identified PDB IDs containing Spike-bound polypeptide TEs (as of November 27, 2021), identified polypeptide TEs, modeled complexes, and consensus-scored complexes. A distinct TE can be represented in more than one PDB ID and some PDB IDs include multiple distinct TEs or multiple instances of a single TE bound to Spike. Each instance was considered a separate complex (i.e., TE-Spike interface) for the scope of this study.

To begin to shed light on these questions at the atomic level in 3D and aid experimental characterization and potential redesign of therapeutic entities (TEs), we turned to the wealth of experimental structures of variant Spike-TE interactions generated by the structural biology community and freely available from the Protein Data Bank (PDB) (8), along with recent advances in artificial intelligence/machine learning–based protein structure prediction (9–11). These valuable resources can be critical in understanding and even optimizing the immune response against SARS-CoV-2 variants; for example, our group has recently used a structure-centered approach to identify and report an immunogenic epitope that remains universal across all major emergent variants (12, 13). Here, we describe the generation and characterization of computed structural models (CSMs) of the Omicron VOC (B.1.1.529) Spike and that of 2 subvariants (BA.2.12.1 and BA.5) in the unbound and bound states with 282 distinct polypeptide TEs (including antibodies, nanobodies/synbodies, and other polypeptides) (Figure 2A). Consensus scores for interface energetic changes based on these CSMs were calculated for 735 Spike-TE complexes (Figure 2B). Alterations in individual Spike-TE interfaces and a large-scale analysis of these complexes provide molecular insights into the mechanisms for dampened immune response in Omicron and its recently emerged subvariants and suggest avenues for redesign of TEs (e.g., antibody combinations) as effective countermeasures against the Omicron VOC and its emerging subvariants.

We surmise that making these CSMs widely available will aid experimental research efforts studying Spike-antibody recognition in generating hypotheses for altered recognition over the evolution of the Omicron VOC and its subvariants, and enable further characterization and even redesign of therapeutically relevant complexes. Given that SARS-CoV-2 Spike recognition is central to biochemical interactions between our immune systems and the virus, and the current unmet need to understand the efficacy of immune response against novel Omicron subvariants, we hypothesize that large-scale, structure-based analyses of Spike interfaces would provide key qualitative insights and structural knowledge. We are, therefore, sharing these CSMs with the scientific and medical research communities at large. As such, all of the CSMs together with associated summary sheets described in this work are available online (https://github.com/sagark101/omicron_models).

## Results

### Analysis of B.1.1.529, BA.2.12.1, and BA.5 Spike-ACE2 binding interface.

**Figure 3:**
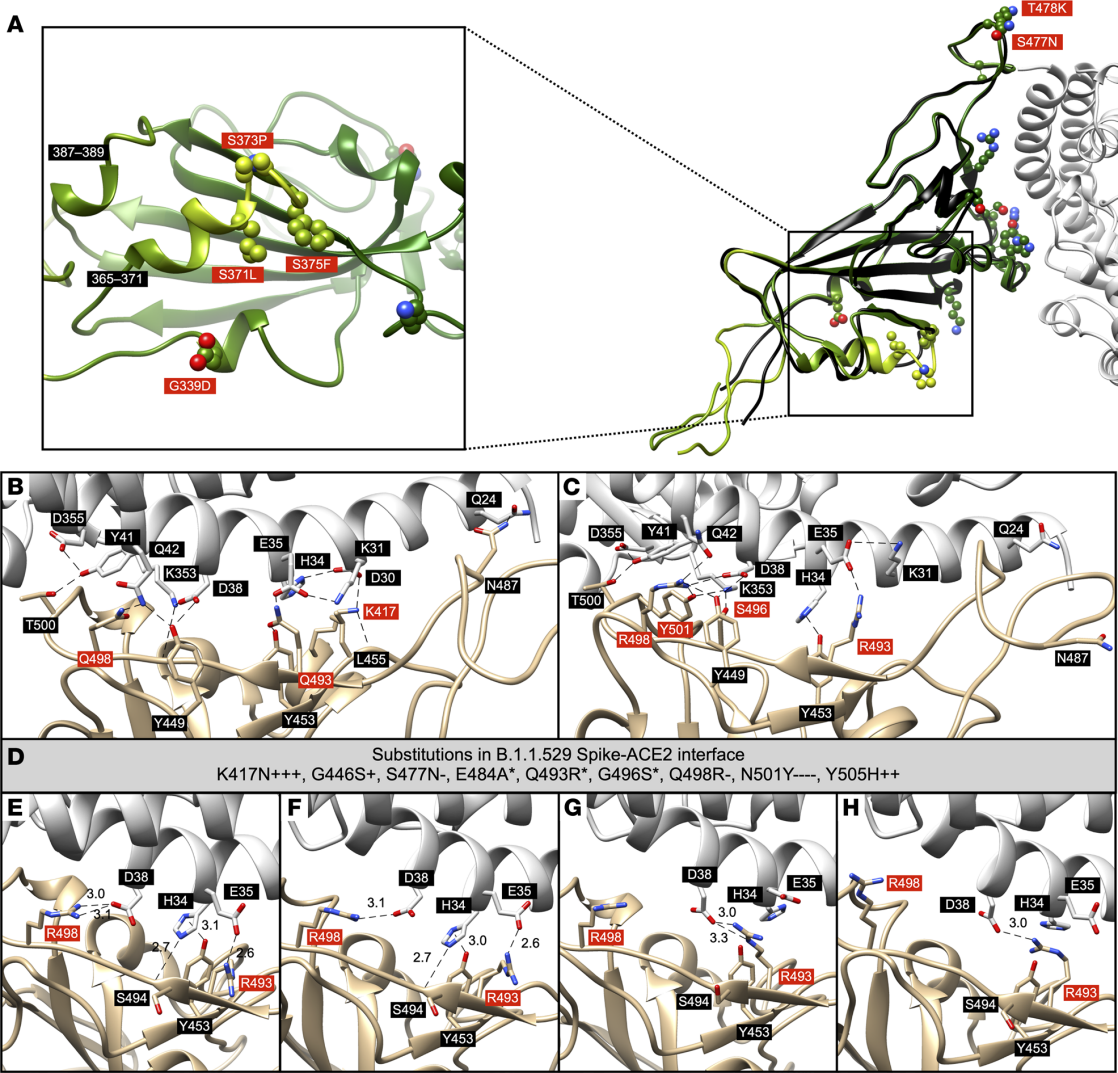
Comparisons between experimentally determined structures and CSMs of the B.1.1.529 RBD–ACE2 complex. (**A**) AFRC model of B.1.1.529 RBD in complex with ACE2 (light gray) (PDB ID: 7EKG). The cryo-EM structure of B.1.1.529 RBD (black) (PDB ID: 7T9L) is overlaid with modeled B.1.1.529 RBD. Modeled B.1.1.529 RBD residues are colored by AF2-reported pLDDT scores (yellow-to-green spectrum corresponds to low-to-high confidence). Inset: Substituted residues with low pLDDT scores in comparison with other substitutions are highlighted (red labeling). (**B**) Previously determined x-ray crystal structure of wild-type RBD bound to ACE2 (PDB ID: 6M0J). (**C**) Previously determined cryo-EM structure of B.1.1.529 RBD bound to ACE2 (PDB ID: 7T9L). (**D**) Substituted residues of Spike involved in the binding interface and their relative energy changes across all CSMs (RRMC, RRMF, AFRC, AFRF) of B.1.1.529 RBD bound to ACE2 based on available PDB IDs. “+” indicates destabilization and “–” indicates stabilization (absolute magnitude larger than 1.4 REU). Number of “+” or “–” symbols indicates our confidence in the prediction (3 or 4: high, 2: moderate, 1: low); * indicates a situation where there are conflicting predictions from 2 or more methods. (**E**–**H**) Overview of key interactions predicted correctly (**E** and **F**) (RRMC and RRMF models of PDB ID: 7EKG) or incorrectly (**G** and **H**) (AFRC and RRMC models of PDB ID: 6M0J) based on the experimentally determined structure of B.1.1.529 RBD-ACE2. Length unit of noncovalent bonds (dotted lines) between Spike and ACE2 is given in ångströms.

To investigate the impact of RBD residue substitutions on ACE2 binding, we examined the CSMs of B.1.1.529 RBD bound to ACE2 and compared them to experimental structures of the wild-type RBD-ACE2 complex and B.1.1.529 RBD-ACE2 complex (N.B., no experimental structures exist for BA.2.12.1 or BA.5 RBD-ACE2) as a benchmark for validity (Figure 3). To generate the CSMs, we started from established RBD-ACE2 structures of other variants, including wild-type (14), Alpha (15), Beta (15), Gamma (15), P1 (16), Delta (PDB ID: 7V89), and an in vitro–evolved variant (17). All of the structures included a single RBD chain except Delta, which included 3, for a total of 9 Spike-ACE2 complexes.

All of the residue substitutions at the ACE2 interface are accommodated in a favorable geometry and appear to make several favorable intra- and intermolecular contacts, consistent with binding to ACE2 being preserved despite the large number of changes observed in the RBD. Many of the substitutions or sites of substitutions in B.1.1.529 RBD were observed individually in other variants (N501Y, first observed in Alpha and Gamma; K417N and E484K, first observed in Beta; T478K, first observed in Delta). Some of these residue substitutions have been observed in pairwise combinations in RBDs evolved for increasing affinity to the ACE2 receptor (17). Analyses of synonymous and nonsynonymous mutations in the gene encoding the B.1.1.529 Spike compared with previously detected SARS-CoV-2 variants indicate that several key sites of residue substitution have likely undergone positive selection, suggesting that the observed substitutions, particularly those occurring in the receptor-binding motif (RBM), may be evolutionarily beneficial to the virus (18). One key question for a markedly altered VOC such as Omicron is whether or not these individual (presumably) favorable substitutions will interact favorably (positive cooperativity/epistasis) or unfavorably (negative cooperativity/epistasis), and, if so, what might be the structural basis for such an effect? Compared with the wild-type complex (Figure 3B), a cryo–electron microscopy (cryo-EM) structure of B.1.1.529 RBD-ACE2 (PDB ID: 7T9L) (19) documents that for some RBD substitutions that cluster in a single binding region, enthalpically favorable positive cooperativity arises as a result of an extensive interaction network (featuring Y501, R498, S496, and R493 from RBD; D355, Y41, Q42, D38, H34, E35, and K31 from ACE2) between the B.1.1.529 RBD substitutions and ACE2 residues in the binding site (Figure 3C). In this extensive network of interactions, the B.1.1.529 RBD-ACE2 interface bears some resemblance to other evolved protein-protein interfaces with optimized, often interdigitated side-chain–to–side-chain interactions (20, 21).

Consensus scores were calculated based on the CSMs for 9 different Spike-ACE2 Rosetta Repack-Minimize Constrained (RRMC), Rosetta Repack-Minimize Free (RRMF), AlphaFold2 (AF2) Repack-Minimize Constrained (AFRC), and AF2 Repack-Minimize Free (AFRF), and a qualitative destabilization consensus score was calculated that incorporated energetic changes observed in CSMs from the 4 methods. Analysis of calculated energy changes across the B.1.1.529 CSMs based on all other Spike RBD-ACE2 variant complexes in the PDB (Figure 3D) suggests that N501Y contributes favorably to the binding (with high consensus), while K417N leads to the loss of an interface hydrogen bond. However, N417 is involved in 2 intramolecular hydrogen bonds that may increase the stability of the bound conformation. Overall, the B.1.1.529 CSMs reported here suggest that ACE2 binding is robust, with the emergence of a network of interactions.

We compared the CSMs for B.1.1.529 RBD-ACE2 to an experimental structure (PDB ID: 7T9L) as a benchmark of our methodology. Comparison of CSMs generated by using all 4 methods from different RBD-ACE2 structures demonstrates that starting model and modeling method affect whether the final CSM recapitulates specific side-chain interactions or suggests alternative contact networks (Figure 3, E–H); small backbone perturbations may favor or preclude certain rotamer conformations. Most CSMs captured the majority of interactions correctly, although there were exceptions. For instance, in this experimental structure of B.1.1.529 RBD-ACE2, R493 of RBD binds E35 of ACE2 and Y453 binds H34 of ACE2 (Figure 3C). The 7EKG RRMC model captured the experimental interactions correctly (Figure 3E). However, the 6M0J AFRC model instead had R493 of B.1.1.529 RBD bound to D38 of ACE2 (Figure 3G). As another example, in this experimental structure, R498 has a hydrogen-bond network that includes N501Y, G496S, and Y449 of B.1.1.529 RBD, and D38 and Q41 of ACE2 (Figure 3C). The 7EKG RRMF model captures most of these interactions (Figure 3F), whereas the 6M0J RRMC model includes none (Figure 3H). We compared key metrics of the RRM and AFR models of B.1.1.529 RBD bound to ACE2 (mean ± SD); we found that the RRM models demonstrate lower root-mean-square deviation (RMSD) values compared with the aforementioned experimental structures of B.1.1.529 RBD bound to ACE2 (PDB IDs: 7T9L and 7T9K) (1.7 ± 0.4 vs. 2.9 ± 0.7 Å) than AF2 models do, since the former use experimentally determined RBD-ACE2 structures of other variants as starting points. However, the AF2 models had greater intermolecular hydrogen bond counts (5.4 ± 1.5 vs. 5.3 ± 1.9) and lower interfacial energy (–43.2 ± 7.3 vs. –41.9 ± 6.5 REU) (Supplemental Table 6; supplemental material available online with this article; https://doi.org/10.1172/jci.insight.168296DS1). This variability highlights the value of considering structural diversity, which we do in this study by including both RRM and AFR models.

Additional recently published structures of SARS-CoV-2 Omicron Spike complexed with human ACE2 (PDB IDs: 7U0N, 7WBP, 7WBL, 7XCI, and 7TN0) include differences in the side-chains forming interfacial contacts (22–25). For example, Y449, S496, and/or Y501 do not make any interfacial polar interactions in some experimental structures (PDB IDs: 7WBL, 7TN0, 7WBP, and 7U0N). Furthermore, 7XCI does not demonstrate any interfacial polar/charged interactions with Y449, R493, S496, R498, and Y501 of the RBD. Given the variability of these experimentally determined structures, we infer that the interface naturally assumes an ensemble of conformational states, of which our CSMs may be members. Hence, it is possible that the different interfacial conformations observed in the CSMs may occur as part of normal molecular conformational diversity. To summarize, we were able to recapitulate a number of experimentally observed interactions and the differences that we predicted are plausible, thereby validating our modeling pipeline and highlighting the importance of sampling diverse structures for each RBD-binding entity complex for obtaining predictions that match experimental data.

**Figure 4:**
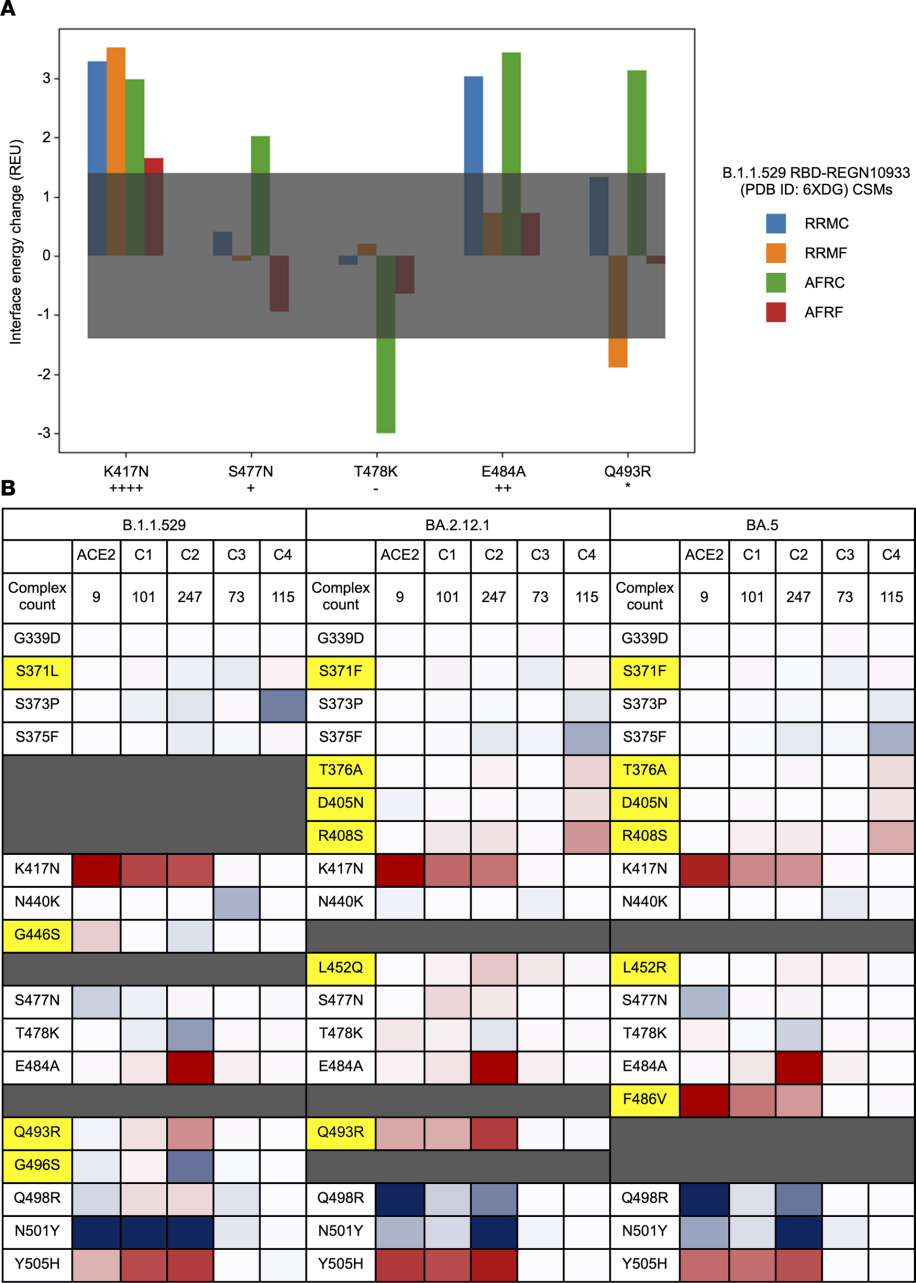
Consensus scoring of energetic effects on binding to ACE2 or polypeptide TEs by substituted residue of Spike RBD across B.1.1.529, BA.2.12.1, and BA.5. (**A**) Per-residue interface scores (in Rosetta energy units) at substituted residue sites for REGN10933 (PDB ID: 6XDG) modeled with B.1.1.529 as an illustrative example of consensus scoring. Colored bars represent the 4 different modeling methods. The gray box represents the 1.4-REU threshold for counting as stabilizing/destabilizing. In this case, the consensus string, with overall consensus score contributions shown in parenthesis, is K417N++++ (+1), S477N+ (+0.125), T478K– (–0.125), E484A++ (+0.25), and Q493R* (+0). The complex consensus score is +1.25. (**B**) Energy-based consensus scoring by residue substitution represented as heatmaps for RBD-bound ACE2 in addition to antibodies, nanobodies/synbodies, and other polypeptide TEs by Barnes class (C1, C2, C3, and C4) for B.1.1.529, BA.2.12.1, and BA.5. Consensus scores are totaled for each site across all models with ACE2 or a TE of a given class. Coloration scale is normalized to the model count for each class, with red indicating overall destabilization and blue indicating overall stabilization. Cells with darker shades indicate greater overall stabilization or destabilization. Substitutions highlighted in yellow indicate residue substitutions that are inconsistent across B.1.1.529, BA.2.12.1, and BA.5.

We present the relative energy changes associated with all RBD-substituted residues in the context of interaction with ACE2 for B.1.1.529 in addition to BA.2.12.1 and BA.5 in Figure 4. BA.2.12.1 and BA.5 do not demonstrate major differences within the Spike-ACE2 interface in comparison to B.1.1.529. Additional substituted residues at sites 376, 405, 408, and 452 are distant from the interface and thus result in minimal energetic impact. However, notable impacts for BA.2.12.1 and BA.5 include the reversions of G446S and G496S, which have destabilizing and neutral effects for B.1.1.529, respectively.

### Analysis of B.1.1.529, BA.2.12.1, and BA.5 Spike-TE binding interfaces.

One of the plausible scenarios advanced to explain the origin of the Omicron VOC is long-term circulation in an immunocompromised individual (presumably human) with persistent SARS-CoV-2 infection (26). Within such an environmental niche, it may be reasonably posited that the RBD has accrued substitutions endowing it with both increased infectivity and immune-escape attributes. Indeed, several sites of residue substitutions in the Omicron VOC have been observed in other variants that exhibit dampened antibody binding (27, 28) and in yeast-based mapping deep mutational escape experiments carried out with a large set of antibodies (29–31). These previous results initially suggested that the Omicron VOC is capable of immune evasion from neutralizing and therapeutic antibodies (31). As this VOC and its subvariants appear to have many escape-endowing residue substitutions, a key question is whether their individual effects are additive or cooperative (i.e., synergistic or antagonistic). In other words, can the weakened affinity due to one substitution be compensated by additional interactions due to another substitution? Because these interactions are inherently 3D structure–mediated phenomena, we reasoned that CSMs of B.1.1.529, BA.2.12.1, and BA.5 Spike-TE complexes may provide insights into possible interplay between the effects of individual residue substitutions over the course of the evolving Omicron VOC.

Polypeptide TEs that target the RBD of SARS-CoV-2 Spike have been divided by Barnes et al. (32) into 4 classes (Barnes classes), depending on whether their binding sites overlap with the ACE2-binding site and the orientation of RBD for accessibility (up or down). Briefly: (i) Class 1 antibodies have epitopes on Spike that overlap with the ACE2-binding site and can only bind to RBD in the up (open) conformation (e.g., C102, C105, B38, CC12.3, COVOX-222, and COVOX-253). (ii) Class 2 antibodies are characterized by Spike epitopes that overlap with the ACE2-binding site and can bind RBD irrespective of its conformation (up/open or down/closed) (e.g., C002, C104, C119, C121, C144, COVA2-39, 5A6, P2B-2F6, Ab2-4, and BD23). (iii) Class 3 antibodies bind the RBD in either conformation (up/open or down/closed) at an epitope distal to the ACE2-binding site (e.g., C110, C135, REGN10987, and S309). (iv) Class 4 antibodies are capable of binding the RBD only in its up/open conformation at an epitope that does not overlap with the ACE2-binding site (e.g., CR3022, COV1-16, EY6A, S304, and S2A4).

We generated CSMs of B.1.1.529, BA.2.12.1, and BA.5 Spike bound to 282 unique Spike-bound TEs. In particular, we generated CSMs of 735 complexes with both Rosetta and AF2, and 95 complexes with only AF2. The utilized Rosetta protocol only samples minor backbone changes that might result from residue substitutions and therefore would not produce reliable structure predictions for insertions or deletions. Hence, it was not feasible to perform Rosetta modeling or consensus evaluation for complexes involving the N-terminal domain (NTD) within the scope of this study. The numerical breakdown of unique polypeptide TEs and complexes for which CSMs were generated is listed in Table 2.

Energy-based consensus scores from the CSMs were calculated across all RBD-TE complexes (including antibodies, nanobodies/synbodies, and other polypeptide TEs), summarized in Figure 4 and provided on per-TE and per-complex bases in Supplemental Tables 7 and 8 (which include data for all unique polypeptide TEs, some of which target sites other than RBD, in this study). In total, 735 TE complexes (i.e., 101 class 1, 247 class 2, 73 class 3, 115 class 4, and 199 unclassified) were consensus scored (Table 2). Of note, consensus scoring requires Rosetta- and AF2-based CSMs. Therefore, consensus scores were not calculated for complexes of TEs binding to the NTD. Furthermore, consensus scoring was not performed for 1 class 3 TE complex (antibody G32R7, PDB ID: 7N64) (33) and 1 class 4 TE complex (nanobody/synbody n3113, PDB ID: 7VNE) (34) due to an interface containing the NTD in addition to the RBD. Overall, for the RBD-binding TEs assigned to a Barnes class, we observe greater destabilization in class 1 and class 2 antibodies, with greatest destabilizations arising from K417N, E484A, and Y505H, which are common to B.1.1.529, BA.2.12.1, and BA.5. For B.1.1.529 and BA.2.12.1, Q493R also demonstrates noteworthy destabilization, while for BA.5, F486V elicits a notably high degree of destabilization. Overall, we see less destabilization in antibodies of class 3 and class 4, but the trend for greater destabilization by subvariants of Omicron is consistent. Furthermore, similar trends were noted between heatmaps subdivided by AFR and RRM models (Supplemental Figure 1), further supporting general agreement between Rosetta- and AF2-based approaches.

**Figure 5:**
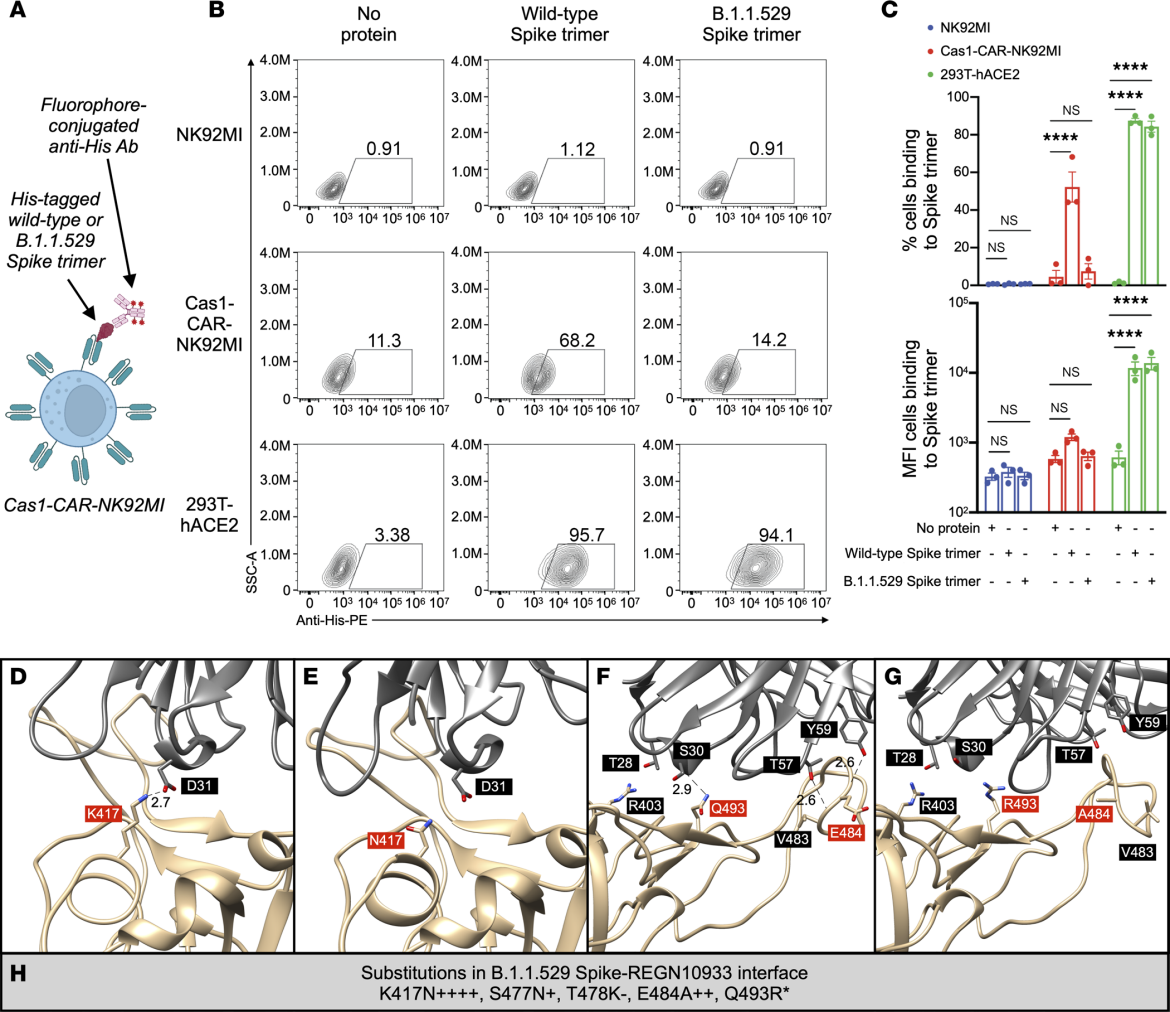
Experimental and computational data reveal that REGN10933 has lower affinity for B.1.1.529 Spike than wild-type Spike. (**A**) Schematic of the Cas1-CAR-NK92MI cells binding to recombinant His-tagged Spike trimer. Illustrations were generated with BioRender. (**B**) Representative contour plots demonstrating the binding of Cas1-CAR-NK92MI cells (displaying REGN1033 as a scFv-CAR on NK92MI cell surface) to wild-type Spike trimer but not B.1.1.529 Spike trimer (as percentage of cells). (**C**) Quantitative binding efficiency of Cas1-CAR-NK92MI cells in both percentage and mean fluorescence intensity (MFI). Each dot represents 1 independent experiment (*n* = 3; analyzed with 2-way ANOVA and post hoc Dunnett’s multiple-comparison test; NS, *P* > 0.05; ****P < 0.0001). Data shown as mean ± SEM. (**D**–**H**) Key substituted residues (red) in the interface between B.1.1.529 Spike (tan) and REGN10933 (dim gray) that undergo notable energy changes based on its (**D** and **E**) AFRF and (**F** and **G**) RRMC models (PDB ID: 6XDG). Length unit of noncovalent bonds (dotted lines) between Spike and antibody is in ångströms. (**H**) Substituted residues of B.1.1.529 Spike involved in the binding interface with REGN10933 and their relative energy changes across 4 predicted models (RRMC, RRMF, AFRC, AFRF). Number of “+” or “–” symbols indicates our confidence in the prediction (3 or 4: high, 2: moderate, 1: low); * indicates a situation where there are conflicting predictions from 2 or more methods.

We sought to evaluate the binding between B.1.1.529 Spike and a clinically relevant class 1 antibody experimentally to assess the validity of our computational approach. In particular, we investigated REGN10933 (casirivimab), which is part of the REGEN-COV (formerly known as REGN-COV2) combination (from Regeneron Pharmaceuticals) along with REGN10987 (imdevimab) (35–37). Thus, we performed a casirivimab–chimeric antigen receptor (CAR)–NK (hereafter referred to as Cas1-CAR-NK) cell binding assay. Cas1-CAR-NK cells displaying REGN10933 as a single chain variable fragment (scFv) were generated to achieve a transduction efficiency of 52.2%. Cas1-CAR-NK cells were subsequently used for a Spike trimer binding assay to test the efficiency of binding to wild-type and B.1.1.529 Spike trimers (Figure 5A). By using NK92MI as a negative control and 293T-hACE2 as a positive control, we demonstrate a markedly higher binding efficiency of Cas1-CAR-NK92MI cells to wild-type Spike trimer compared with that of B.1.1.529 Spike trimer (Figure 5, B and C). Loss of this interaction may be explained at the atomic level according to the CSMs (Figure 5, D–G), which demonstrate loss of key interactions and hence destabilization at the RBD-REGN10933 interface (Figure 5H). K417N may result in a salt bridge loss (Figure 5, D and E), while E484A and Q493R may lead to loss of hydrogen bonds (Figure 5, F and G). In other studies, the following fold reductions have been seen experimentally for each binding mutation to REGN10933 according to the Stanford University Coronavirus Antiviral & Resistance Database (CoV-RDB) (27, 28): K417N (4.4- to >100-fold), S477N (0.9- to 3.4-fold), and Q493R (21- to 70-fold). In the CSMs, destabilizing energy changes corresponding to these substitutions are observed (Figure 5H), and no compensatory interactions are evident in any of the CSMs. Altogether, these results are consistent with findings from other experimental studies, demonstrating the validity of our streamlined approach (38–40).

**Figure 6:**
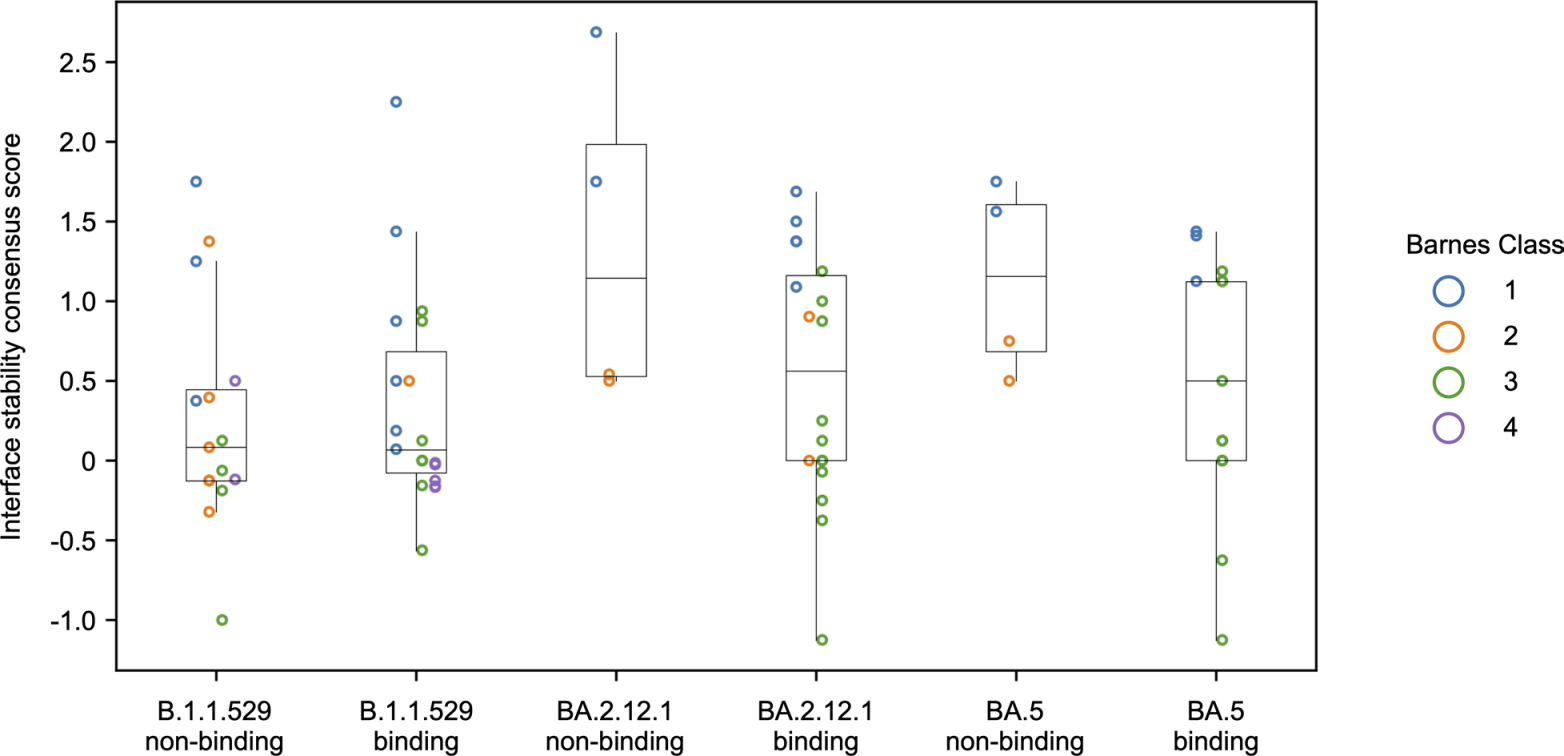
Interface stability consensus scores for B.1.1.529, BA.2.12.1, and BA.5 RBD binding to TEs by Barnes class. Each point represents 1 experimentally tested TE, colored by Barnes class, and showing calculated consensus score, with positive values predicting lessened affinity and negative or near-zero values predicting retained affinity. Box-and-whisker plots exclude outliers (see Methods).

Finally, we aimed to assess the level of noise in the consensus-based predictions by confirming that positive consensus scores correlate with experimentally observed reductions in affinity. By using experimental data from CoV-AbDab (41) and CoV-RDB (27, 28), we identified antibodies that had been individually experimentally assayed for binding activity on the 3 modeled strains. Any antibody identified as having weak or strong binding in CoV-AbDab or a 100-fold or less change in CoV-RDB was classified as binding. A notable limitation to this approach is that it allows a binary identification of whether binding was lost, but does not identify where affinity is weakened but not lost; thus, the expected correlation between our predictions and the experimental determinations is not expected to be quantitative. We identified 34 TEs tested on B.1.1.529 (15 nonbinding, 19 binding), 22 tested on BA.2.12.1 (4 nonbinding, 18 binding), and 13 tested on BA.5 (4 nonbinding, 13 binding) (Figure 6 and Supplemental Table 9). The expected pattern appeared for BA.2.12.1 and BA.5, wherein stability consensus scores tended to be higher (destabilizing) for nonbinding TEs, and binding TEs tended to have lower predicted destabilization. Nonbinding B.1.1.529 TEs had a tendency toward lower destabilization in general, compared with nonbinding TEs for the other strains. These results indicate that the generated CSMs appropriately predicted overall stabilization or destabilization of Spike-TE interactions with respect to known experimental findings.

## Discussion

The emergence, spread, and evolution of the Omicron VOC of SARS-CoV-2 has raised several concerns regarding transmissibility, severity of disease, and susceptibility to existing antibodies. To bolster research efforts aimed at prospectively characterizing new VOCs and their subvariants, we generated CSMs of Spike of the original SARS-CoV-2 Omicron VOC (B.1.1.529) and its subvariants, BA.2.12.1 and BA.5, bound to ACE2 and diverse antibodies, nanobodies/synbodies, and other polypeptide TEs by using streamlined computational methods, including AF2-based structure prediction and Rosetta-based energy calculations. Based solely on these CSMs, we assessed the qualitative impact of substituted residues within the interface between B.1.1.529, BA.2.12.1, and BA.5 Spike and various binding partners to facilitate greater understanding at a molecular level of how these substitutions may impact SARS-CoV-2 infection and antibody neutralization.

Analyses of B.1.1.529, BA.2.12.1, and BA.5 RBD interactions with various proteins shed light on how the relatively numerous residue substitutions in these globally emerging SARS-CoV-2 variants may exert their effects individually and exhibit cooperativity. ACE2 binding by B.1.1.529 RBD appears to involve a highly interdigitated network of interactions, suggesting a structural basis for cooperativity (or positive epistasis; Figure 3). This is largely consistent with experimental findings of this complex (19). Binding of B.1.1.529, BA.2.12.1, and BA.5 RBD to class 1 and 2 TEs appears to be considerably affected, with destabilizing interactions dominating over a small number of stabilizing interactions arising from the numerous substitutions in the Omicron VOC and its 2 subvariants. On the other hand, interaction networks in class 3 and class 4 TEs are only modestly affected. In total, we have generated CSMs and analyses for 282 unique polypeptide TEs (which also include TEs bound to Spike at sites other than the RBD), encompassing 835 complexes, of which 735 were modeled using all 4 modeling approaches (RRMC, RRMF, AFRC, and AFRM) and consensus scored. A comprehensive qualitative picture of the impact of variant substitutions on the binding properties of SARS-CoV-2 Spike has emerged from this study, in agreement with other experimental data (19).

We anticipate that the CSMs of Spike complexes that we have made available will be useful for other global research efforts. First, for experimental investigators interested in characterizing the impact of individual or clusters of substitutions observed in Spike, the CSMs provide visual aids plus many testable hypotheses. Networks of interactions identified for ACE2 binding, for example (Figure 3), can be tested by using site-directed mutagenesis (30, 42). In cases where the binding of therapeutic antibodies is functionally compromised, these CSMs may provide starting points for computer-aided antibody redesign efforts. Thus, the CSMs reported herein provide atomic-level insights into ligand-directed 3D recognition of B.1.1.529, BA.2.12.1, and BA.5 RBD by various binding proteins and TEs.

Several limitations and caveats apply to the CSMs and analyses presented here. First, posttranslational modifications such as glycosylation, which may often be a key element of Spike structure, are not considered in the protein-only models. Many experimental structure determinations also exclude these glycans, and multiple x-ray structures show that the ACE2-binding interface does not explicitly involve glycans. However, some glycosylation patterns do affect binding of certain antibodies. Second, we do not include explicit interfacial water molecules in the modeling procedures. There were no major water-mediated interactions observed experimentally in the complexes we analyzed in detail. However, it is conceivable that newly established water-mediated interactions have been missed. Both limitations can be addressed by using the CSMs reported here as starting points for more computationally expensive modeling approaches (such as molecular dynamics simulations) with appropriately glycosylated amino acid side-chains (43).

In addition to these limitations inherent to modeling procedures, there are well-known limitations of the energy functions used for scoring and energy evaluations plus the limited conformational sampling that is practically performed in the interest of computational efficiency. Because the overall structures of B.1.1.529, BA.2.12.1, and BA.5 Spike are highly similar to wild-type Spike, limited sampling around the conformations observed in experimentally derived structures represents a reasonable approximation. We have, however, explicitly explored alternative conformations by using both AF2- and experimentally derived structures to generate small ensembles of similar structures as starting points of our analyses, and assign confidence based on consensus within the ensemble. AF2 generates folded protein structures given a variant sequence, though recent literature has indicated that physics-based modeling may be more accurate for a small number of substitutions (44–46) and our own results demonstrated higher RMSDs than RRM modeling. Furthermore, although AF2 produced reasonable models for individual protein domains, it did not successfully produce multimeric complexes with the correct relative orientations of the component proteins; thus, our AF2 models required a physics-based minimization after superposition. We addressed the uncertainty as to which methods were most suitable for 15–17 substitutions in the RBD, with consideration for the possibility that no one method might be the most suitable for all complexes in the study, and opted to use both modeling approaches and take a consensus. Thus, we believe that a reasonable balance has been struck between conformational sampling and computational expediency, and we found that AF2- and Rosetta-based models were largely in agreement across the set of modeled TEs (Supplemental Figure 1). The structures and predictions provided in this work may serve as a starting point, supplement, or guide for other researchers studying the mutational consequences of Omicron and other as yet uncharacterized SARS-CoV-2 VOCs.

In summary, we have organized relevant PDB data and generated CSMs of Omicron VOC and 2 subvariant Spike complexes with ACE2 and nearly all experimentally defined polypeptide TEs, including antibodies from all 4 Barnes classes (32). Based on these CSMs, we observe strikingly altered interface topologies in the computed protein-protein interfaces. We made qualitative inferences about the energetic impact of substituted residues occurring within interfaces to provide hypotheses regarding their effects on abrogation and structural remodeling of interactions. We have made these data available (https://github.com/sagark101/omicron_models) for the broad scientific community to complement ongoing research, and hope that they will be of use to experimental hypothesis testing, therapeutic antibody design, and other efforts to mitigate the global response to the SARS-CoV-2 pandemic.

Although public cautionary measures have relaxed with the passage of time and improvements in treatment, SARS-CoV-2 remains a potentially lethal threat, especially to those who are immunocompromised. The ability to rapidly screen a library of antibodies for maintained or reduced efficacy upon identification of a new strain, or the ability to select efficacious antibodies upon sequencing a patient’s infecting virus, may be invaluable. New strains continue to arise, such as XBB.1.5 (47), which has several novel mutation sites (R346T, L368I, V445P, N460K, F490S) and substitutions at previously observed sites (G339H, F486P), and appears to demonstrate the continued ability of the virus to evolve immune evasion. In addition to providing a framework for rapid response to the identification of these novel strains, we hope that our methods, or other similar high-throughput evaluations of changes in ACE2 and TE affinities upon mutation, might be used to forecast novel strains and prepare against them.

## Methods

### Overview.

With the goal of evaluating changes in ACE2 and TE affinity that result from Spike substitutions present in SARS-CoV-2 Omicron VOC and subvariants, we first aimed to produce reasonable CSMs of the Spike complexes using Rosetta- and AF2-based approaches (Figure 2A). We used experimentally determined structures as a starting point, most of which were produced before the rise of any VOCs, and then computationally applied the substitutions of the variants in our study. We also chose to model both with and without coordinate constraints, which would reduce deviation from the starting backbone coordinates (the original strain in the case of Rosetta-refined experimental structures and the solution-state model in the case of AF2 structures). Thus, we had 4 methods for modeling: Rosetta modeling of variants from experimental structures with and without constraints, and AF2-generated variant RBDs that were superimposed into complex with respective binding partners, and then minimized using Rosetta with and without constraints. For each structure produced by these 4 methods, we calculated per-residue energetic changes for all substitutions, and compared the values calculated for each to determine a qualitative prediction consensus of affinity change.

### Curation of SARS-CoV-2 Spike structures in the PDB.

We curated structures of Spike bound to polypeptide TEs (388 distinct PDB IDs), which encompass 286 unique polypeptide TEs (Figure 2B). All Spike-bound TEs were subdivided into 3 possible groups: antibody, nanobody/synbody, and other. The combination status of PDB IDs was determined (a combination is 2 or more unique bound proteins in a single multiprotein complex). Bound proteins/peptides were listed for each PDB ID and, if present in combination, specifically by polypeptide chain(s). Furthermore, RBD-bound polypeptide TEs were grouped by Barnes classification according to the CoV3D database (48) and/or literature (32, 49, 50) if available.

### AF2 modeling of Spike of Omicron VOC and subvariants.

We used AF2 to generate CSMs of RBD and various other Spike fragments (S1 domain, full monomer, full trimer). We modeled full B.1.1.529, BA.2.12.1, and BA.5 Spike sequences as monomers with AF2 and attempted to model trimers as well. Predicted structures of individual domains and the Spike monomer were deemed credible. The overall predicted local-distance difference (pLDDT) scores, ranging from 0–100, are generally higher than 75 for the top models of monomers and more than 85 for the top models of the individual domains. A score above 70 for a predicted structure was considered to be in the confident range (11, 51). The AF2-based computational process used here predicts 5 structural models and ranks them according to their pLDDT scores, and the highest-scoring model was used (11, 51). Confidence in predicted structure is deemed to be high when the score is greater than 90, medium when the score is between 70 and 90, low when the score is less than 70, and very low when the score is less than 50. Pairwise RMSD values comparing C_α_ atomic positions for experimental structures of several other variants’ Spike fall in the range of 0.3–1.3 Å (Supplemental Table 1). We have further benchmarked the AF2-predicted monomers by comparison with several other variant Spike experimental structures in the PDB, including Alpha, Beta, Gamma, Delta, and P1 variants, and a variant RBD produced by directed evolution (17). C_α_ atom RMSD values range from 0.4–0.8 Å (Supplemental Table 2).

AF2-predicted single-domain models of the B.1.1.529, BA.2.12.1, and BA.5 RBDs are also similar to Rosetta-minimized PDB-based structures of B.1.1.529, BA.2.12.1, and BA.5 RBDs bound to various entities. C_α_ atom RMSD values range from 0.5–2.2 Å (Supplemental Table 3), which is comparable to the structural variation observed within the set of experimental Spike structures. N- and C-termini are typically less well-structured in both experimental and predicted models, and, therefore, represent a substantial source of the structural deviations. Thus, according to the pLDDT scores that AF2 assigns to each residue (Figure 3A and Supplemental Figure 2), there is high confidence in the portion of the B.1.1.529 RBD that interacts with ACE2, and lower confidence closer to both termini. This finding is consistent with the known flexibility of Spike in this region, which allows the RBD to hinge between open and closed conformations. The greatest structural prediction uncertainty within the B.1.1.529 RBD polypeptide chain occurs in the region of residues 364–376, in which 3 sites are substituted in the Omicron VOC (Figure 3A, inset). Notably, among these substitutions is S373P, which would be expected to cause disruption or kinking of the helix compared with wild-type. It should also be noted that these residues have more positive (i.e., unfavorable) scores in the Rosetta-computed models, indicating a similar lack of confidence in the correct folding around those substituted residues. Finally, when assessing binding involving the regions with low-confidence predictions, one should recognize that the low confidence of the unbound structure propagates into lower confidence in the energetic calculations at those sites.

### Variant modeling approach.

We have previously developed an in silico mutagenesis and analysis pipeline for studying the ongoing populational coevolution of SARS-CoV-2 (52). In our pipeline, residue substitutions observed in sequenced SARS-CoV-2 genomes obtained from the GISAID database (53, 54) are introduced in available PDB structures or CSMs of proteins and their energetic impact is qualitatively evaluated. We have previously focused on evaluating how observed mutations affect the structure and stability of individual proteins and domains for SARS-CoV-2 (52). We adapted this pipeline (Figure 2B) for the evaluation of B.1.1.529, BA.2.12.1, and BA.5 Spike in complex with ACE2 and bound TEs.

### Complex model generation.

To increase computational efficiency during modeling, structures were truncated to include only binding regions of the modeled binding partners. TE-binding regions of Spike were identified with reference to the Structural Antibody Database (SAbDAb) when available (Supplemental Tables 4 and 5) (55). We used 2 starting points for generating models: (i) atomic coordinates of TE-binding regions available in experimental structures archived in the PDB, and (ii) AF2 models of the free TE-binding region, which were superimposed on the atomic coordinates of the TE-binding region in PDB structures. For each type of starting structure, we applied 2 conformational sampling approaches to obtain Rosetta energy-minimized models of the complexes. Interface side-chain rotamers were repacked and gradient-based minimization of the Rosetta-calculated energy was performed with and without positional coordinate restraints on the protein backbone atoms, leading to 4 CSMs for each modeled complex with a given variant. We refer to these CSMs as follows: RRMC, Rosetta Repack-Minimize Constrained; RRMF, Rosetta Repack-Minimize Free; AFRC, AF2 Repack-Minimize Constrained; and AFRF, AF2 Repack-Minimize Free. Repack-Minimize is performed using the Rosetta energy function in all cases (56). AFR modeling used whole-domain structures, so 1 wild-type model was used as base comparison for all 3 variants. RRM modeling optimized a certain set of side-chains, which barely differed between variants. For this reason, each variant had its own RRM wild-type reference models to ensure that all residue-level comparisons had consistent optimization. Having CSMs generated with various degrees of conformational constraints during optimization is intended to provide representative low-energy conformations from the native ensemble of the complex, as our conformational sampling approach involves a tradeoff between computational speed and extensive evaluation of the native ensemble. In total, CSMs were generated for the 282 distinct Spike-bound polypeptide TEs identified in the 388 PDB IDs (Table 2). The remaining 4 TEs (i.e., B6, S2P6, Fab22, EK1) (PDB IDs: 7M53, 7RNJ, 7S3N, 7C53) of the 286 could not be modeled due to incomplete Spike structures at the binding interface.

### Consensus scoring.

With 20 CSMs (RRMC and RRMF variant CSM and wild-type control CSM each for B.1.1.529, BA.2.12.1, and BA.5 Spike, and AFRC and AFRF each for wild-type, B.1.1.529, BA.2.12.1, and BA.5 Spike) for each binding partner in hand, we next examined these CSMs for altered interaction patterns and evaluated per-residue energies of interaction to identify the consensus effect of substitutions. Examination of energy changes across all 4 types of CSMs serves as a measure of confidence in the identified interaction patterns when comparing wild-type Spike with B.1.1.529, BA.2.12.1, or BA.5 Spike. For each CSM, we have used a threshold value of 1.4 Rosetta energy units (REU) to identify energetic changes. For each residue, one “+” is assigned per CSM in which the interface destabilization energy due to substitution exceeds +1.4 REU, and one “–” is assigned per CSM in which it is below –1.4 REU, with + and – canceling each other, and neither is assigned for CSMs where the absolute value of interface energy change was below 1.4 REU. For example, a particular mutation is scored as ++++ (high-consensus destabilizing) if all 4 CSMs involve calculated interface energy increases larger than 1.4 REU. A score of ++ is assigned if 2 of the 4 CSMs feature destabilization greater than the threshold value and 2 others do not have significant energy changes with absolute value greater than 1.4 REU, or if 3 are above 1.4 REU and 1 is below –1.4 REU. A “*” is assigned to a substitution when 2 or more CSMs predict conflicting but substantial changes, indicating low consensus in the prediction. Heats/colors were assigned as a weighted sum with an exponential scale (i.e., we weighted each ++++ as contributing +1, +++ as +0.5, ++ as +0.25, and + as +0.125, and similar negative values for corresponding stabilizing residues). When collected across multiple CSMs, heats are averaged across the total number of CSMs. As cautionary notes, these observations reflect the consequences to substituted residues only, and may or may not directly capture effects in the spatially proximate segments of polypeptide chains; these effects may also contribute to the changes in overall binding energy upon substitution to some extent. More information can be found in the Supplemental Material.

### Generation of Cas1-CAR-NK92MI cells.

The DNA sequence of the scFv domain of casirivimab (REGN10933) (Cas1) was codon-optimized by GENEWIZ. The sequence was cloned into an SFG vector by using FastDigest SalI (Thermo Fisher Scientific, FD0644) and FastDigest XhoI (Thermo Fisher Scientific, FD0694) restriction enzymes. The method for the generation of Cas1-CAR-NK92MI cells has previously been described (57). In brief, HEK293T cells (American Type Culture Collection, CRL-3216) were transfected with a retroviral packaging system. The retrovirus supernatant was collected after 48 hours, filtered, and transduced into NK92MI cells (American Type Culture Collection, CRL-2408) in a RetroNectin-coated 24-well plate. Cas1-CAR-NK92MI cells were cultured in MEMα media containing 12.5% heat-inactivated horse serum, 12.5% fetal bovine serum, 2 mM L-glutamine, 0.02 mM folic acid, 0.1 mM β-mercaptoethanol, 1.5 g/L sodium bicarbonate, and 0.2 mM inositol.

### Cas1-CAR-NK cell binding assay.

The binding assay has previously been described (58). Briefly, wild-type, parental NK92MI or Cas1-CAR-NK92MI or 293T-hACE2 cells (a gift from Abraham Pinter, Public Health Research Institute, Rutgers New Jersey Medical School) were incubated with recombinant His-tagged wild-type Spike trimer (AcroBiosystems, SPN-C52H8), His-tagged B.1.1.529 Spike trimer (AcroBiosystems, SPN-C52Hz), or no protein as a negative control in phosphate-buffered saline (PBS) containing 0.5 mM CaCl_2_ and 0.9 mM MgCl_2_ for 1 hour at 4°C under gentle agitation. Cells were washed with PBS and stained with Alexa Fluor 647 AffiniPure goat anti–human IgG (H+L) (Jackson ImmunoResearch, 109606088) and PE anti-His antibody (clone J095G46, BioLegend, 362603) followed by standard flow cytometry.

### Statistics.

Data in contour plots are shown as percentage values of cells binding to Spike (Figure 5B). Data for the quantitative binding efficiency of Cas1-CAR-NK92MI cells are shown as mean ± standard error of mean (SEM) in both percentage and mean fluorescence intensity (MFI); 2-way ANOVA and post hoc Dunnett’s multiple-comparison test (with multiplicity-adjusted *P* values), where *P* less than 0.05 was deemed statistically significant, were performed by using GraphPad Prism 9 (Figure 5C). Box-and-whisker plots were graphed by using the Matplotlib Python library and exclude outliers so that the upper whisker extends to the last data point less than (Q_3_ + 1.5 × [interquartile range]) and the lower whisker extends to the first data point greater than (Q_1_ – 1.5 × [interquartile range]) (Figure 6). Boxes represent the median (horizontal line) and interquartile range (box boundaries).

### Data availability.

Data are available in the “Supporting data values” XLS file, at https://github.com/sagark101/omicron_models, or from the corresponding authors upon request.

## Author contributions

RP, WA, SKB, and SDK conceptualized and conceived the study. JHL and CM retrieved and organized relevant PDB data. JHL and DB developed computational approaches for generating structural models. JHL, CM, DB, and SDK analyzed structural models. CM visualized structural models. MTM, CHC, and DL performed experimental characterization of antibody binding. JHL, CM, DB, MTM, and SDK wrote the first draft of the manuscript, with subsequent editorial contributions from all other authors. DL, RP, WA, SKB, and SDK directed the overall project. DL, RP, WA, SKB, and SDK funded the project. The order of co–first authors (JHL and CM) was determined by alphabetical order of their last names.

## Supplementary Material

Supplemental data

Supporting data values

## Figures and Tables

**Table 1:**
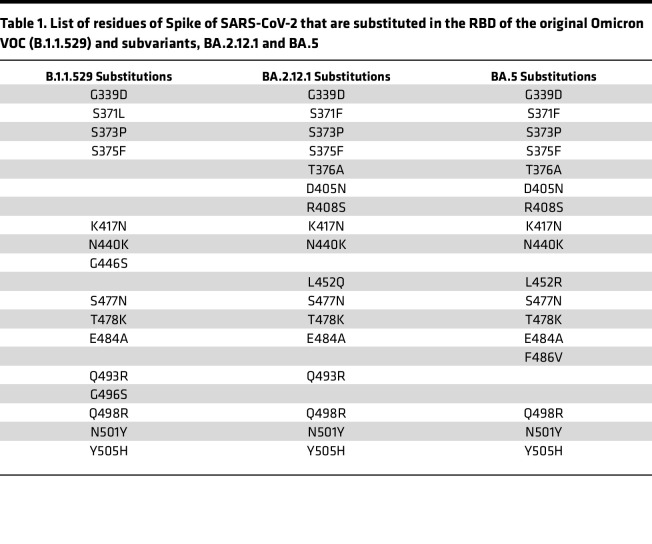
List of residues of Spike of SARS-CoV-2 that are substituted in the RBD of the original Omicron VOC (B.1.1.529) and subvariants, BA.2.12.1 and BA.5

**Table 2:**
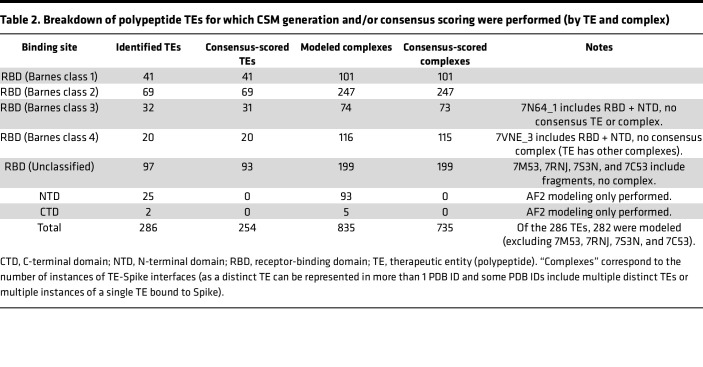
Breakdown of polypeptide TEs for which CSM generation and/or consensus scoring were performed (by TE and complex)

## Version 1 06/01/2023

In-Press Preview

## Version 2 07/10/2023

Electronic publication
