# Supplementary material for: Modeling of ACE2 and antibodies bound to SARS-CoV-2 provides insights into infectivity and immune evasion
Source: JCI Insight. 2023 Jul 10;8(13):e168296. doi: 10.1172/jci.insight.168296 (PMC10371346; doi:10.1172/jci.insight.168296)
Supplement: Supplemental data [file jciinsight-8-168296-s240.pdf]

Supplemental Material for:

Modeling of ACE2 and antibodies bound to SARS-CoV-2 provides insights into infectivity and immune evasion

Joseph H. Lubin[1,*], Christopher Markosian[2,3,*], D. Balamurugan[4,5], Minh T. Ma[6,7], Chih-Hsiung Chen[6,7], Dongfang Liu[6,7], Renata Pasqualini[2,3], Wadih Arap[2,8], Stephen K. Burley[1,9,10,11,12], and Sagar D. Khare[1,11,12]

[1] Department of Chemistry and Chemical Biology, Rutgers, The State University of New Jersey, Piscataway, NJ 08854, USA

[2] Rutgers Cancer Institute of New Jersey, Newark, NJ 07101, USA

[3] Division of Cancer Biology, Department of Radiation Oncology, Rutgers New Jersey Medical School, Newark, NJ 07103, USA

[4] Office of Advanced Research Computing, Rutgers, The State University of New Jersey, Piscataway, NJ 08854, USA

[5] Department of Radiology, Rutgers New Jersey Medical School, Newark, NJ 07103, USA

[6] Department of Pathology, Immunology, and Laboratory Medicine, Rutgers New Jersey Medical School, Newark, NJ 07103, USA

[7] Center for Immunity and Inflammation, Rutgers New Jersey Medical School, Newark, NJ 07103, USA

[8] Division of Hematology/Oncology, Department of Medicine, Rutgers New Jersey Medical School, Newark, NJ 07103, USA

[9] RCSB Protein Data Bank, Rutgers, The State University of New Jersey, Piscataway, NJ 08854, USA

[10] RCSB Protein Data Bank, San Diego Supercomputer Center and Skaggs School of Pharmacy and Pharmaceutical Sciences, University of California San Diego, La Jolla, CA 92067, USA

[11] Rutgers Cancer Institute of New Jersey, New Brunswick, NJ 08901, USA

[12] Institute for Quantitative Biomedicine, Rutgers, The State University of New Jersey, Piscataway, NJ 08854, USA

* Co-first authorship

Model Trimming. Protein Data Bank (PDB) models for antibodies in SAbDab (1) were trimmed to a single Fab (amino acid residues up to 120) and the bound region of the SARS-CoV-2 Spike protein (hereafter referred to as Spike). The Spike regions were defined as N-terminal domain (NTD): residues 14–305; receptor-binding domain (RBD): residues 319–541. Determination of whether to include one or both regions in the trimmed model were based on the detection of interfacial residues contacted by the complementarity-determining region (CDR) loops of the antibody, as defined in SAbDab. Detection of interfacial residues defined a Spike residue as interfacing with the CDR if the C_α atom is within 5.5 Å of any CDR C_α, or within 9 Å of any CDR C_α and the C_α-C_β vector of that residue pair is within 75°. Trimmed models could include two Spike chains if that contact was specified in SAbDab. For uniformity, all chains were renamed so that Spike chains were A (and B, if multiple Spike chains were present) and antibody chains were H and L as identified in SAbDab. Original chain names are listed in **Supplemental Table 4**. The trimmed models were considered sufficient to model the energetic consequences at the binding interface, while removing significant amounts of computational optimization that would not elucidate those consequences. All trimmed models were minimized using the Rosetta FastRelax protocol (2) with coordinate constraints restricting backbone movement prior to other modeling steps explained below. Ten decoys were generated and the single lowest-scoring one (based on total score) was used.

Rosetta Repack-Minimization Modeling. Repack-minimization models were produced using a FastRelax protocol. The backbone was mobile, and the sidechains of all residues that are mutated in Omicron Variant of Concern (VOC) (B.1.1.529) and its sub-variants (BA.2.12.1 and BA.5), as well as all interfacial residues (detected as described in the previous paragraph) with them, were optimized. In the wild-type (*i.e.*, Wuhan-Hu-1) model, the sidechains were optimized with their original sequence in the PDB. In the case of the Omicron models, the sidechains that were optimized were those corresponding to the mutated sequence. This modeling was done in two ways. In the case of constrained models, a score penalty was applied to inhibit significant C_α movement from the starting model. In the free models, no such constraints were

applied, and the optimization scoring was determined purely based on the Rosetta default energy function (3). Ten decoys were generated and the single lowest-scoring one (based on total score) was used in analyses.

AlphaFold2 Modeling. To produce AlphaFold2 (AF2) Spike-therapeutic entity (TE) structures, the best-ranked wild-type and Omicron RBD models were superimposed with the RBD in relaxed trimmed models, and PDB IDs were generated using the existing TE structure with the exchanged RBD. Models were then re-minimized using Rosetta FastRelax, again both with and without constraints. Ten decoys were generated and the single lowest-scoring one (based on total score) was used in analyses.

Energy Calculations. Twenty models for each Spike-TE complex, between the two modeling methods, Rosetta Repack-Minimize (RRM) and AF2 Repack-Minimize (AFR), each used both with and without positional restraints, modeling both wild-type and Omicron (B.1.1.529, BA.2.12.1, and BA.5) Spikes. Interfacial energies were calculated as the sum of pairwise energies across an interface (either single-residue with TE or full Spike structure with TE).

References

1. Dunbar J, Krawczyk K, Leem J, Baker T, Fuchs A, Georges G, et al. SAbDab: The structural antibody database. *Nucleic Acids Research.* 2013;42(D1):D1140-D6.
2. Nivon LG, Moretti R, and Baker D. A Pareto-optimal refinement method for protein design scaffolds. *PLoS One.* 2013;8(4):e59004.
3. Alford RF, Leaver-Fay A, Jeliazkov JR, O'Meara MJ, DiMaio FP, Park H, et al. The Rosetta all-atom energy function for macromolecular modeling and design. *J Chem Theory Comput.* 2017;13(6):3031-48.
4. Gowthaman R, Guest JD, Yin R, Adolf-Bryfogle J, Schief WR, and Pierce BG. CoV3D: A database of high resolution coronavirus protein structures. *Nucleic Acids Res.* 2021;49(D1):D282-D7.

Supplemental Table 1: Benchmark comparisons between the AF2-predicted full-sequence B.1.1.529 RBD and several other wild-type Spike structures in complexes.

PDB ID	C_α RMSD (Å)
6VXX	0.56
6XC4	0.54
6XCM	0.81
6XDG	0.90
6YLA	0.43
6ZDH	0.57
6ZGE	0.81
7BEP	0.41
7K8S	0.66
7K8X	0.71
7KMG	0.37
7LRT	1.01
7M7W	0.50
7MM0	1.29
7NX6	0.54
7ORA	0.34
7R6X	0.40

Supplemental Table 2: Benchmark comparisons between the AF2-predicted full-sequence B.1.1.529 Spike monomer and several variant Spike structures in the PDB.

PDB ID	C_α RMSD (Å)
7EKF (Alpha)	0.38
7EKF (Beta)	0.36
7EKC (Gamma)	0.37
7V89 (Delta)	0.79
7NXC (P1)	0.41
7BH9 (Evolved RBD)	0.52

Supplemental Table 3: Benchmark comparisons between the RBD structure of the AF2-predicted B.1.1.529 Spike monomer and the RBD structures in the TE-bound complexes used in our analyses.

PDB ID	Complex	Wild-Type C_α RMSD (Å)	B.1.1.529 C_α RMSD (Å)
6M0J	ACE2	1.133	1.161
6XC4	1	0.601	0.611
6XC4	2	0.647	0.634
6XCM	1	0.997	1.044
6XCM	2	0.901	0.99
6XDG	1	1.027	1.073
6XDG	2	1.25	1.368
6YLA	1	0.724	0.709
6YLA	2	0.701	0.688
6ZDH	1	0.943	1.033
6ZDH	2	0.912	0.903
6ZDH	3	0.74	0.712
7BEP	1	0.607	0.652
7BEP	2	0.667	0.815
7BEP	3	0.606	0.7
7BEP	4	0.636	0.719
7K8S	1	1.246	1.256
7K8S	2	1.448	1.47
7K8S	3	1.233	1.199
7K8X	1	1.814	1.659
7K8X	2	0.928	0.89
7KMG	1	0.596	0.73
7KMG	2	0.659	0.783
7LRT	1	1.462	1.4
7M7W	1	0.757	0.867
7M7W	2	0.728	0.825
7M7W	3	0.653	0.697
7M7W	4	0.685	0.715
7MM0	1	2.159	2.18
7NX6	1	0.611	0.653
7NX6	2	0.618	0.644
7ORA	1	0.514	0.516
7ORA	2	0.515	0.534
7ORA	3	0.575	0.648
7ORA	4	0.59	0.652
7R6X	1	0.643	0.75
7R6X	2	0.632	0.67
7R6X	3	0.621	0.652

Supplemental Table 4: pdb_complexes.csv (https://github.com/sagark101/omicron_models/blob/main/pdb_complexes.csv)
Identification of PDB ID chains and domains extracted into each trimmed model and used for subsequent CSM generation.

Supplemental Table 5: TEs_by_PDB.csv (https://github.com/sagark101/omicron_models/blob/main/TEs_by_PDB.csv)

PDB IDs with identified TEs. PDB IDs including a combination with multiple different antibodies/spike-binding protein types are split into separate rows for each type. The Model Name column identifies the corresponding structures in trimmed_pdb_structures (https://github.com/sagark101/omicron_models/tree/main/trimmed_pdb_structures) and CSMs (https://github.com/sagark101/omicron_models/tree/main/CSMs) folders. Notes may indicate TE molecule type or the presence of mutations in the baseline RBD model. They are also noted if the CoV3D database (4) differs from other available literature, and if so, what class the database indicates. Several PDB IDs are excluded, either because they did not include significant portions of the spike protein in complex with polypeptide TEs; such PDB IDs have 'X' in the Model Name column.

Supplemental Table 6: Structural analysis of RRM and AFR models of B.1.1.529 RBD bound to ACE2 (number of hydrogen bonds, interfacial energy, and RMSD in comparison to baseline experimental structures).

CSM	starting_model	method	constraint	base_strain	HBonds	int_E	RMSD_7T9L_1	RMSD_7T9K_1	RMSD_7T9K_2
6M0J_1_rpk_cons_om	6M0J_1	RRM	cons	WT	5	-41.9971	1.30592	1.373552	1.369322
6M0J_1_rpk_free_om	6M0J_1	RRM	free	WT	3	-47.2733	1.391136	1.452898	1.445336
6M0J_1_af2_cons_om	6M0J_1	AFR	cons	WT	4	-39.7219	1.639799	3.023993	3.059548
6M0J_1_af2_free_om	6M0J_1	AFR	free	WT	6	-46.7233	2.568871	3.805301	3.842996
7EKF_1_rpk_cons_om	7EKF_1	RRM	cons	Alpha	4	-38.412	1.349478	1.408269	1.403528
7EKF_1_rpk_free_om	7EKF_1	RRM	free	Alpha	7	-51.7016	1.478001	1.546466	1.540499
7EKF_1_af2_cons_om	7EKF_1	AFR	cons	Alpha	5	-43.0351	1.694272	3.127855	3.16552
7EKF_1_af2_free_om	7EKF_1	AFR	free	Alpha	6	-53.4549	2.169889	3.490501	3.522993
7EKG_1_rpk_cons_om	7EKG_1	RRM	cons	Beta	4	-42.5181	1.370345	1.432188	1.427649
7EKG_1_rpk_free_om	7EKG_1	RRM	free	Beta	5	-47.179	1.586105	1.655388	1.650878
7EKG_1_af2_cons_om	7EKG_1	AFR	cons	Beta	5	-42.6725	1.696811	3.103017	3.139944
7EKG_1_af2_free_om	7EKG_1	AFR	free	Beta	6	-43.2317	2.176895	3.742167	3.792615
7EKC_1_rpk_cons_om	7EKC_1	RRM	cons	Gamma	2	-38.7709	1.392964	1.452013	1.447822
7EKC_1_rpk_free_om	7EKC_1	RRM	free	Gamma	7	-49.8938	1.523591	1.584619	1.583756
7EKC_1_af2_cons_om	7EKC_1	AFR	cons	Gamma	5	-45.3065	1.735026	3.125118	3.161296
7EKC_1_af2_free_om	7EKC_1	AFR	free	Gamma	6	-43.6803	2.350669	3.740875	3.781279
7V89_1_rpk_cons_om	7V89_1	RRM	cons	Delta	8	-35.7082	1.915308	2.01476	2.037831
7V89_1_rpk_free_om	7V89_1	RRM	free	Delta	8	-44.22	2.346488	2.45297	2.467036
7V89_1_af2_cons_om	7V89_1	AFR	cons	Delta	3	-33.8464	2.056481	3.249725	3.289195
7V89_1_af2_free_om	7V89_1	AFR	free	Delta	7	-48.2149	2.083744	2.999795	3.020681
7V89_2_rpk_cons_om	7V89_2	RRM	cons	Delta	6	-30.3153	1.876379	1.984047	2.010008
7V89_2_rpk_free_om	7V89_2	RRM	free	Delta	8	-45.9326	2.291068	2.446512	2.424288
7V89_2_af2_cons_om	7V89_2	AFR	cons	Delta	3	-27.306	2.018644	3.237453	3.278181
7V89_2_af2_free_om	7V89_2	AFR	free	Delta	7	-51.5531	2.249706	3.07796	3.110049
7V89_3_rpk_cons_om	7V89_3	RRM	cons	Delta	5	-31.2666	1.893709	2.000616	2.031003
7V89_3_rpk_free_om	7V89_3	RRM	free	Delta	5	-41.047	2.350979	2.504147	2.498402
7V89_3_af2_cons_om	7V89_3	AFR	cons	Delta	3	-31.0962	2.030483	3.229399	3.268506
7V89_3_af2_free_om	7V89_3	AFR	free	Delta	7	-48.7268	2.610622	3.821799	3.846205
7NXC_1_rpk_cons_om	7NXC_1	RRM	cons	P1	3	-43.4807	1.301956	1.361618	1.358459
7NXC_1_rpk_free_om	7NXC_1	RRM	free	P1	6	-43.8185	1.363389	1.409928	1.406786
7NXC_1_af2_cons_om	7NXC_1	AFR	cons	P1	5	-44.7295	1.737853	3.035479	3.069179
7NXC_1_af2_free_om	7NXC_1	AFR	free	P1	7	-44.8565	2.422793	3.682413	3.714132
7BH9_1_rpk_cons_om	7BH9_1	RRM	cons	Evolved	3	-31.2559	1.603295	1.633295	1.625035
7BH9_1_rpk_free_om	7BH9_1	RRM	free	Evolved	7	-48.7697	1.738355	1.805046	1.801036
7BH9_1_af2_cons_om	7BH9_1	AFR	cons	Evolved	5	-35.9151	1.968836	3.201654	3.236979
7BH9_1_af2_free_om	7BH9_1	AFR	free	Evolved	8	-53.2054	1.818349	2.697298	2.707699

Supplemental Table 7: TE_class_complexes_consensus.xlsx (https://github.com/sagark101/omicron_models/blob/main/TE_class_complexes_consensus.xlsx) Polypeptide TEs with associated PDB complexes. Total consensus scores are included for each variant, averaged across all complexes of a given TE. Score cells are colored according to score, with red indicating destabilization and blue indicating stabilization. Several TEs are excluded, due to available PDB IDs not including significant portions of the spike protein in complex with the TE; such TEs have 0 in the Complex Count column.

Supplemental Table 8: TE_consensus_by_complex.xlsx (https://github.com/sagark101/omicron_models/blob/main/TE_consensus_by_complex.xlsx)
The granular data aggregated to produce TE_class_complexes_consensus.xlsx and Figure 4. Per-residue consensus information for each RBD-binding complex is listed.

Supplemental Table 9: heats_vs_expt.csv (https://github.com/sagark101/omicron_models/blob/main/heats_vs_expt.csv)

Heat and experimental data used to generate Figure 6.

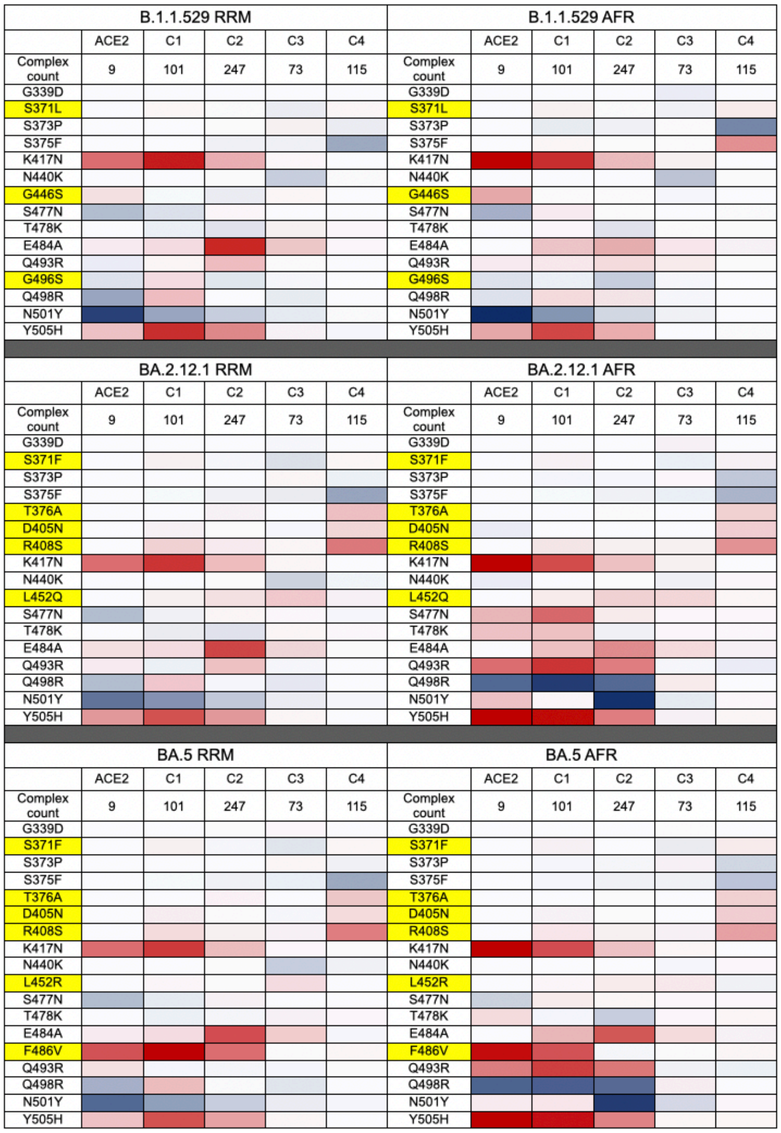

Supplemental Figure 1: Energy-based consensus scoring by residue substitution represented as heat maps for RBD-bound ACE2 in addition to antibodies, nanobodies/ synbodies, and other polypeptide TEs by Barnes Class (C1, C2, C3, and C4) for B.1.1.529, BA.2.12.1, and BA.5 separated by RRM and AFR models. Consensus scores are totaled for each site across all models with ACE2 or a TE of a given class. Coloration scale is normalized to the model count for each class, with red indicating overall destabilization and blue indicating overall stabilization. Cells with darker shades indicate greater overall stabilization or destabilization. Substitutions highlighted in yellow indicate residue substitutions that are inconsistent across B.1.1.529, BA.2.12.1, and BA.5. Data were combined to generate Figure 4B.

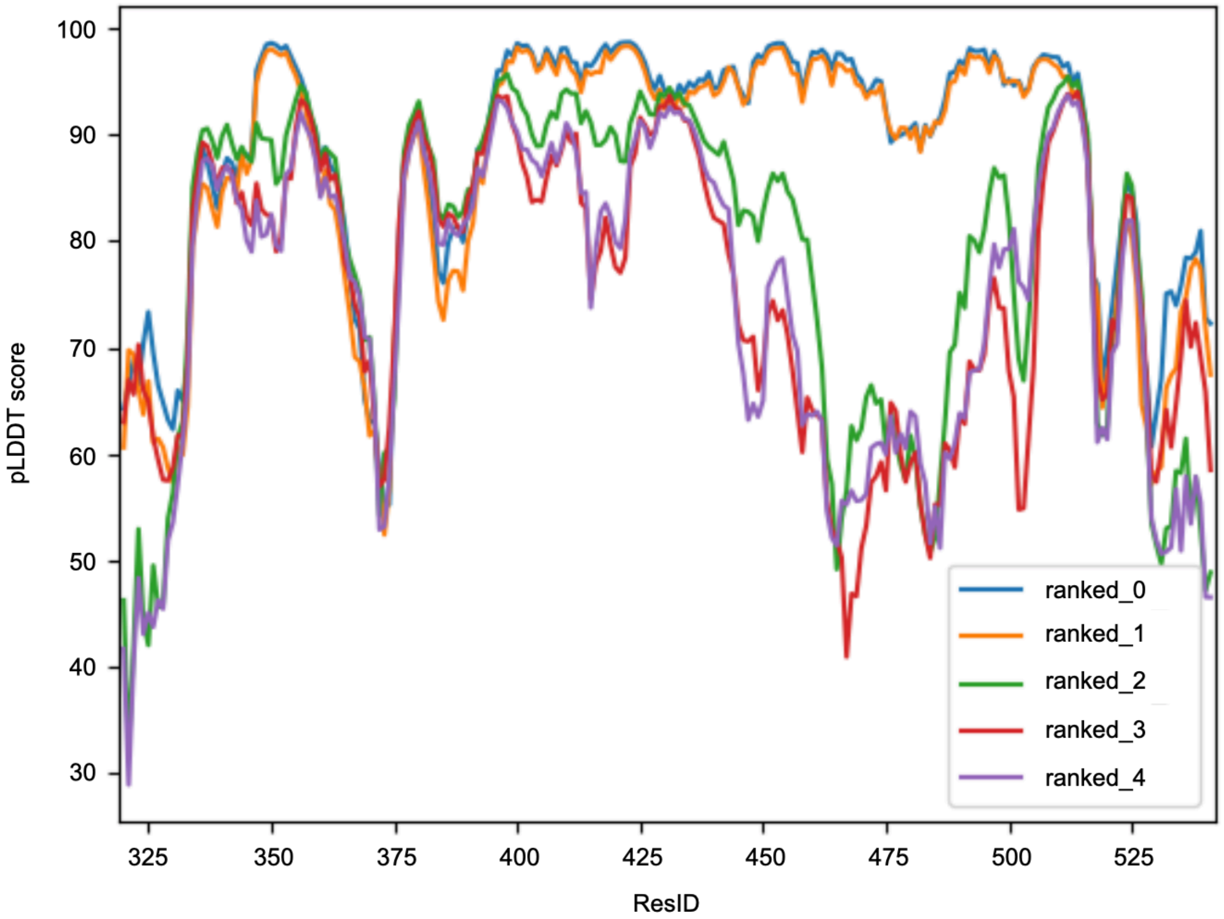

Supplemental Figure 2: pLDDT score of each residue within B.1.1.529 RBD for the five AF2-predicted models. Residues (ResIDs) of isolated B.1.1.529 RBD are numbered according to their positions relative to wild-type Spike.

Supplemental CSMs: CSMs (https://github.com/sagark101/omicron_models/tree/main/CSMs) includes AF2_base_domains (https://github.com/sagark101/omicron_models/tree/main/CSMs/AF2_base_domains), which replaced experimental spike domains for AFR model generation. Directory also includes all representative CSM structures used in the analysis, organized into subfolder by structure type (ACE2 or TE), generation method (AFR or RRM), strain (in the case of RRM models; all AFR strains are grouped), and complex ID. Models were generated for both variant and wild-type reference for complexes involving the RBD, whereas the insertions/deletions in the NTD are only modeled with AF2. AF2 models were generated for all complexes involving the RBD and/or NTD for all strains, and comparison was always to the wild-type.

Other Supplemental Figures and Tables: CSM_analysis (https://github.com/sagark101/omicron_models/tree/main/CSM_analysis)
Figures and tables are organized in subdirectories by structure type (ACE2 or TE), strain, and complex ID.

***_single_res_energies.csv**

Each complex has a table in its respective folder listing the per-residue energy changes for each site in each model. The first four columns identify the TE and modeling method. The following three columns, wt, om, and mutated, indicate the sequence at that site, with mutated being a boolean indication that wt and om (whichever variant is modeled) are not the same. wt_total, om_total, and d_total represent the computed Rosetta total energy for the residue of the wild-type and Omicron models, and the difference between them. wt_interface, om_interface, and d_interface represent the same, but just the sum of pairwise interfacial scores for the RBD residue with all nearby TE residues, rather than the full complex scores. d_interface was used for consensus scoring.

***_total_res_energies.png and *_interface_res_energies.png**

Each complex includes four pairs of line plot figures, corresponding to the four modeling methods (RRMC, RRMF, AFRC, AFRF), comparing per-residue total and interfacial energies in REU between the wild-type and Omicron models. The difference is the subtraction of the first panel (wild-type) from the second (Omicron). If multiple spike chains are included, the numbering for the second chain is +1000. Bars for RRM models are absent for NTD-binding TEs.

***_mutated_interface_res_energies.png and *_non-mutated_interface_res_energies.png**

Each complex includes a pair of bar plot figures indicating the significant residue energy changes. The grey box indicates the ±1.4 REU threshold for significance. Sites with at least one CSM per-residue interface energy exceeding the threshold are included in the figure. Figures are divided between mutated sites and non-mutated sites. Bars for RRM models are absent for NTD-binding TEs. The mutated_interface_res_energies figures are visual representations of the consensus scores in TE_consensus_by_complex.xlsx.
